# Efficient Media for High Lipase Production: One Variable at a Time Approach

**Published:** 2017

**Authors:** Safoura Soleymani, Houri Alizadeh, Hossein Mohammadian, Emad Rabbani, Fatemeh Moazen, Hamid MirMohammad Sadeghi, Ziaedin Samsam Shariat, Zahra Etemadifar, Mohammed Rabbani

**Affiliations:** 1. Department of Pharmaceutical Biotechnology, Isfahan Pharmaceutical Sciences Research Center, Faculty of Pharmacy and Pharmaceutical Sciences, Isfahan University of Medical Sciences, Isfahan, Iran; 2. Department of Biochemistry, Faculty of Pharmacy and Pharmaceutical Sciences, Isfahan University of Medical Sciences, Isfahan, Iran; 3. Department of Biology, Faculty of Basic Sciences, University of Isfahan, Isfahan, Iran

**Keywords:** *Basillus pumilus ZR-5*, Culture media, Lipase enzyme

## Abstract

**Background:**

Lipase enzymes have applications in a wide range of industries. A crucial determining factor of industrial prices of these enzymes is the culture media composition that is constantly under review by researchers. In this work, for maximum lipase production by *Bacillus sp. ZR-5*, culture media compositions were optimized using ″one variable at a time″ strategy.

**Methods:**

For this purpose, the culture medium parameters such as low and high cost carbon and nitrogen sources, substrates and incubation times were evaluated.

**Results:**

Maximum lipase activity was achieved after 24 *hr* of incubation with 1.5% of glucose syrup (1600±69.1 *u/mg*), 1% of fish powder (1238±36.7 *u/mg*) and olive oil (1407±2.1 *u/mg*) as low cost carbon and nitrogen sources and substrate, respectively.

**Conclusion:**

Our results show a significant increase in lipase activity with usage of low cost sources; this could help in reducing the media prices for industrial application of lipase enzyme.

## Introduction

Recent developments in enzymology attracted numerous researchers throughout the world for production of industrial enzymes from microorganisms, especially bacteria, because of their feasibility for large scale production in fermenters. Hydrolytic enzymes such as proteases, amylases and lipases have important role with wide range of applications in many industries particularly food processing, detergent, pharmaceutical, dairy, animal feed and cosmetics ^1–6^.

Lipases are one of the most important classes of hydrolytic enzymes which catalyze hydrolysis of triacylglycerol to diacylglycerol, fatty acids and glycerol. The main substrate of lipase (triacylglycerol hydrolase) is a low water soluble triacylglycerol and glycerol. A wide variety of lipolytic enzymes are produced by microorganism’s mostly bacterial and fungal species. Among these microorganisms, the one which releases the products into extracellular space is more suitable for various reasons. *Bacillus* genus is a well-known source for lipase production due to its ability to secrete a large amount of enzyme in its extracellular space ^1,7–9^. The production of extracellular lipases is influenced by several nutritional and physico-chemical factors such as availability of carbon and nitrogen sources, incubation time, temperature, pH, presence of lipid as an inducer and/or substrate ^1,7,10^.

Great demand in lipase enzymes has forced the scientists to look for new sources of bacteria that can grow in cheaper media conditions. Optimization of medium compositions and use of suitable physico-chemical conditions are vital in biological process in order to achieve a better yield at a low cost. To achieve this, one variable at a time approach was developed by scientists for optimization of production process ^7,11,12^.

The aim of the present study was to optimize the media condition of a bacteria strain that was found in our screening studies of local soil sources and it was named *Bacillus pumilus ZR-5. Bacillus pumilus* can produce a heat stable extracellular lipase with low molecular weight which shows a tendency to short-chain triglycerides ^9,13^. For this purpose, an attempt was made to test the culture medium factors including many low and high cost carbon and nitrogen sources, substrates and incubation times by one variable at a time strategy. Also, the best combinational sources of substrate, carbon and nitrogen on lipase production at the best incubation time were recognized.

## Materials and Methods

### Materials

Rhodamine B, nutrient broth, agar, peptone, yeast extract, 4-nitrophenyl-decanoate, gum Arabic and sodium taurocholate were purchased from Sigma, USA. NaCl, beef extract, Bradford, glucose, fructose, sucrose and malt extract, K_2_HPO_4_, KH_2_PO_4_, CaCl_2_, MgSO_4_.7-H_2_O and (NH_4_)_2_SO_4_ were supplied by Merck, Germany. Starch, molasses and glucose syrup, wheat bran, gluten, dried fish powder, gelatin, corn and soya bean were prepared from the market and or animal feed wholesale.

### Bacterial strain

The bacterial strain used in this study was *Bacillus sp. ZR-5*, that was previously isolated from a bank of Zayande-rood river in Isfahan and identified as *Bacillus pumilus* species. The lipolytic activity of the bacteria was verified by Rhodamine B plate assay. Bacterial cells were cultivated in a Rhodamine B plate containing 8 *g/L* nutrient broth, 4 *g/L* agar, 10 *ml* of Rhodamine B solution (1.0 *mg/ml* distilled water and sterilized) and 31.25 *ml* olive oil at pH=7.0. After incubation at 37°*C* for 48 *hr*, the plate was irradiated with UV light (350 *nm*) ^10^. The orange fluorescence halo indicated lipolytic activity of *Bacillus sp. ZR-5*.

### Culture media composition

Pre-culture medium for *Bacillus sp. ZR-5* was nutrient broth medium containing (*g/L*) peptone ^5^, NaCl ^5^, beef extract ^1^ and yeast extract ^2^. The pre-culture incubation time was 16 *hr* at 37°*C* with shaking at 180 *rpm*. The composition of the basal culture media for lipase production was as follows (*g/L*): glucose (10), peptone (10), yeast extract (5), NaCl (5), K_2_HPO_4_ (0.3), KH_2_PO_4_ (1), CaCl_2_ (2), MgSO_4_.7H_2_O (0.2), (NH_4_)_2_SO_4_ (2) and olive oil 2% (*v/v*). The pH of medium was adjusted to 7.4. In this study, three different concentrations (10, 15 and 2 *g/L*) of four high cost carbon sources including glucose, fructose, sucrose and malt extract were used instead of glucose (10 *g/L*) in basal medium. Three low cost carbon sources were starch, molasses and glucose syrup. Tryptone as a high cost nitrogen source was replaced with peptone (10 *g/L*). Six low cost nitrogen sources including wheat bran, gluten, dried fish powder, gelatin, corn and soya bean were added in place of basal medium nitrogen source. Olive oil in basal medium was taken as a substrate. Glucose (10 *g/L*), peptone (10 *g/L*) and olive oil in basal medium were considered as control groups in carbon and nitrogen sources and substrate, respectively. For measuring enzyme activity in these conditions, 2 *ml* of pre-culture media was inoculated and then the flasks were incubated at 37°*C* under shaking (180 *rpm*). The lipase activity was determined after 24, 48 and 72 *hr* of incubation.

### Lipase activity assay

Lipase activity was determined by measuring the release of 4-nitrophenyl from 4-nitrophenyl-decanoate as a substrate. The reaction mixture contained 395 *μl* of 0.1 *M* sodium phosphate buffer pH=7.4, 90 *μl* substrate (4-NP-decanoate), 25 *μl* gum Arabic, and 50 *μl* sodium taurocholate. The final concentrations were 1 *mM* for substrate, 0.5% for gum Arabic, and 2 *mM* for sodium taurcholate. The mixture was incubated at 37°*C* in water bath for 30 *min*, and then the reaction was started by addition of an aliquot of 50 *μl* crude enzyme solution continued for an additional 30 *min* at 37°*C* ^2,10^. For preparing enzyme of culture medium, 1 *ml* of basal culture was centrifuged for 10 *min* at 10000 *rpm*, 4°*C* and the supernatant as crude enzyme was saved for activity as-says. Absorbance of the resulting yellow colored product was measured at 400 *nm* using ELISA reader. One unit of lipase activity was defined as the amount of enzyme capable of liberating 1 *μmol* of 4-nitrophenyl per minute per milligram protein under the condition of assay. The total protein was measured using Bradford method.

### Statistical analysis

The results of lipase activity are expressed as mean±standard deviation (SD). Every experiment was repeated at least three independent times. All data were analyzed utilizing one-way ANOVA Tukey post test (SPSS software) to determine statistical significance. The level of significance was set at p<0.05.

## Results

### Lipolytic activity detection of Bacillus sp. ZR-5

**Figure 1.**
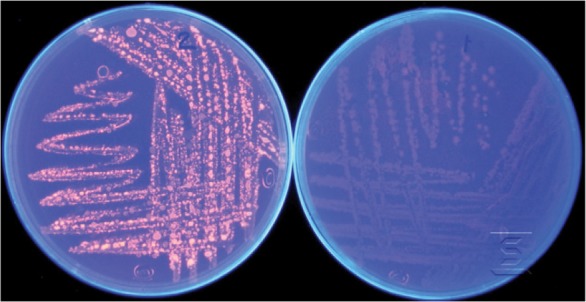
Lipolytic activity detection of *Bacillus sp. ZR-5* with Rhodamin B plate assay test. Orange fluorescent halos around bacterial colonies before (right) and after UV irradiation (left) demonstrate the lipase activity of this strain

Several different methods have been developed for detection of lipolytic activity of microorganisms. In plate assay, lipolytic activity was determined at the presence of the substrates such as tributyrin and olive oil and fluorescent dye Rhodamine B as the indicator. Hydrolysis of substrate leads to the formation of the orange fluorescence zone around bacterial colonies under UV irradiation. Intensity of the orange color increased along with the increase of lipolytic activity ^10,14^. The isolated bacterium *Bacillus sp. ZR-5* was shown to have a lipase activity by producing orange fluorescence halo around the bacterial colonies which hydrolyzed the olive oil substrate (Figure 1). Since the plate assay is a very general method for lipase activity detection, in this study, a faster and more sensitive colorimetric method was applied by using 4-nitrophenyl-decanoate as a substrate.

### Effect of different carbon sources on lipase production

**Figure 2.**
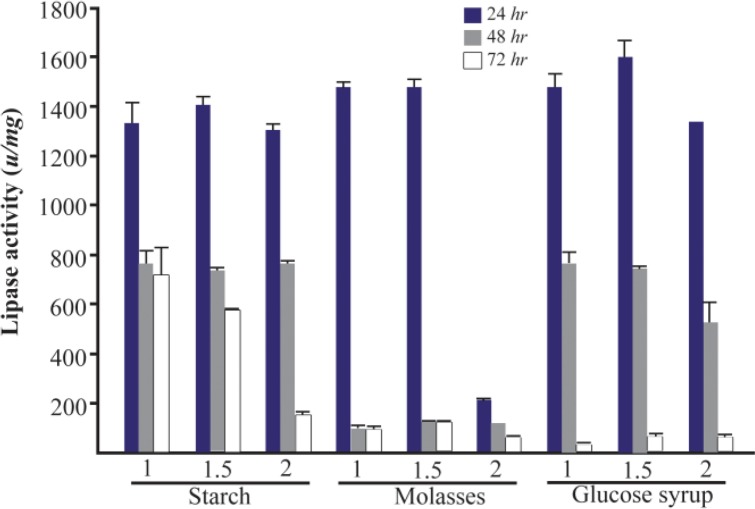
Effect of low cost carbon sources on the activity of lipases at different incubation times (24, 48 and 72 *hr*) by *Bacillus sp. ZR-5*. Molasses 1.5% showed the maximum lipase production among these sources in 24 *hr* (p>0.05).

**Figure 3.**
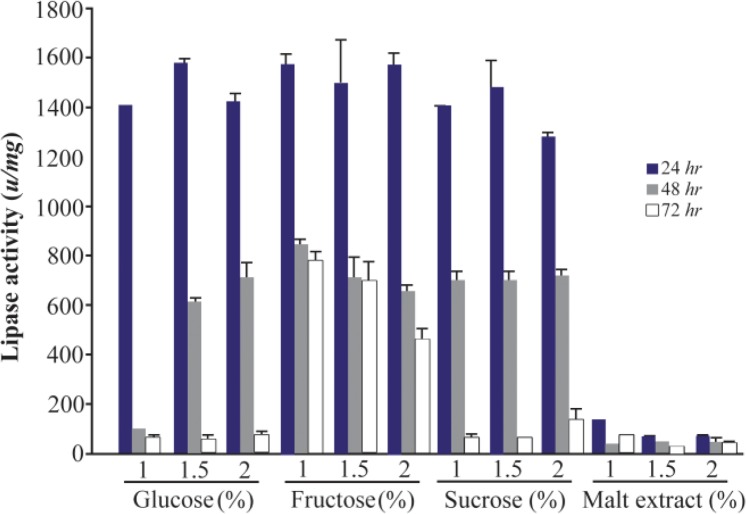
Effect of high cost carbon sources on the activity of lipases at different incubation times (24, 48 and 72 *hr*) by *Bacillus sp. ZR-5*. Glucose 1.5% showed the highest activity in comparison to other sources and concentrations (p>0.05).

It is illustrated that carbon source is a critical factor in the expression of the lipase enzyme ^1^. In this study, for selection of the best carbon source, the lipase activity was measured at the presence of 1, 1.5 and 2% of two groups of low (molasses, glucose syrup and starch) and high cost carbon sources (glucose, fructose, sucrose and malt extract) for three different times (24, 48, and 72 *hr*). Among low cost carbon sources, the lipase activity at 1.5% of glucose syrup was higher than other sources in three concentrations with an activity of 1600±69.1 *u/mg* in 24 *hr* (Figure 2). There were significant differences between the lipase activities in some concentrations of low cost carbon sources (p<0.05). Since molasses has the lowest cost among these sources, molasses (1.5%) with 1478±34.3 *u/mg* lipolytic activity in 24 *hr* was used for further analysis. Between high cost sources, the addition of glucose (1.5%) showed the highest activity (1573±21.6 *u/mg*) in comparison to other sources and concentrations (p>0.05). The lowest lipase activity was obtained by three concentrations of malt extract in 24, 48 and 72 *hr* (Figure 3).

### Effect of different nitrogen sources on lipase production

**Figure 4.**
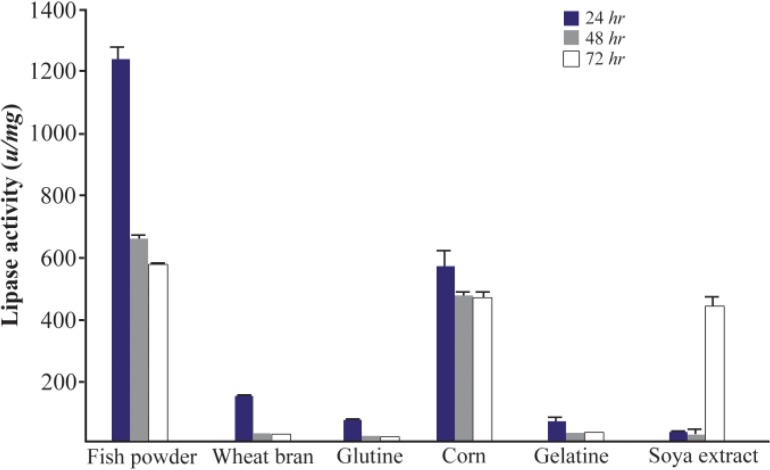
Effect of low cost nitrogen sources on the activity of lipases at different incubation times (24, 48 and 72 *hr*) by *Bacillus sp. ZR-5*. Fish powder at 1% concentration significantly increased the lipase activity after 24 hours of incubation (p<0.05).

**Figure 5.**
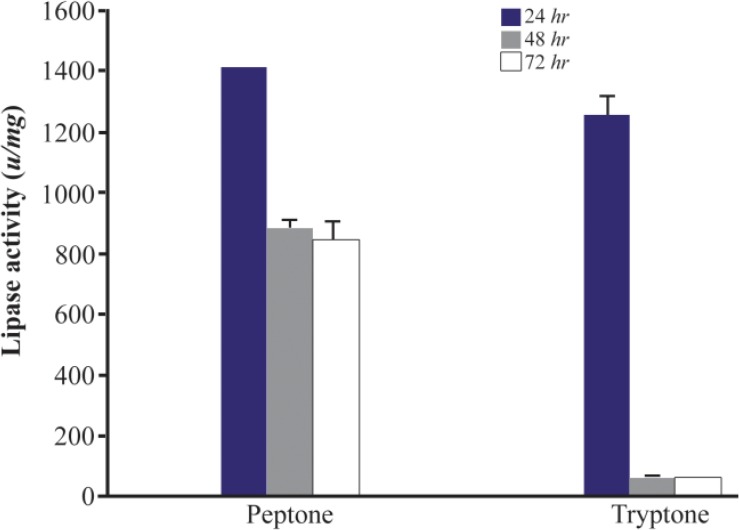
Effect of high cost nitrogen sources on the activity of lipases at different incubation times (24, 48 *hr* and 72 *hr*) by *Bacillus sp. ZR-5*. Peptone showed higher lipase activity than tryptone after 24 *hr* (p<0.05).

Next, the effect of different nitrogen sources on lipase production was measured. The effect of two groups of low and high cost nitrogen sources on the lipase production of *Bacillus sp. ZR-5* bacteria was investigated. Low cost nitrogen sources were wheat bran, gluten, dried fish powder, gelatin, corn and soya bean. Fish powder at 1% concentration significantly increased the lipase activity (1238±36.7 *u/mg*) after 24 *hr* of incubation (p<0.05) (Figure 4). Lipase activity of low cost nitrogen source was close to high cost. Lipase activity of ingluten, gelatin, soya bean and wheat bran was low and therefore, did not significantly change the lipase production (Figure 4). Peptone and tryptone are organic nitrogens that are generally preferred for enzyme production. Our results showed that the lipase activity in the presence of peptone was higher than tryptone with an activity of 1407±2.1 *u/mg* after 24 *hr* (p<0.05) (Figure 5).

### Effect of different substrates on lipase production

**Figure 6.**
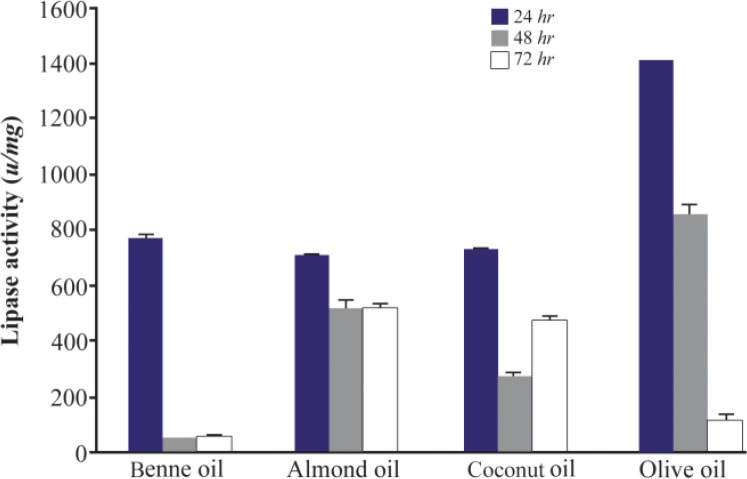
Effect of different substrates on the activity of lipases at different incubation times (24, 48 and 72 *hr*) by *Bacillus sp. ZR-5*. The highest activity was found by olive oil (2%) with an activity after 24 *hr* (p<0.05).

It is demonstrated that lipase enzyme production is generally increased in the presence of oil components. These oils act as a substrate, carbon sources and inducers for lipase production ^1,4^. Bacterial strains used natural oils as an alternative of carbon source when carbon in media was exhausted ^8,15^. In this study, among bean, olive, coconut and almond oils that were tested, the highest activity was found by olive oil (2%) with an activity of 1407±2.1 *u/mg* after 24 *hr* (p<0.05). Lipase activities in the presence of other oils were similar (p>0.05) (Figure 6).

## Discussion

The present study reported on the favorable condition of the lipase enzyme production by *Bacillus sp. ZR-5* using media component optimization. Several important factors were tested for their effects on enzyme yield with the aim of reducing production costs. The results obtained in this work show that the lipase activity for low and high cost carbon source was nearly close to each other. Based on these results, the glucose syrup and molasses were replaced with glucose and fructose. It has been shown that reduction of enzyme activity at higher concentrations of glucose syrup and molasses could be due to the viscosity of the medium component that can be maintained using diluents ^16^. The lipase activity in both groups for all sources was time dependent. The lipase activity reached the maximum value after 24 *hr* of inoculation of *Bacillus sp*. *ZR-5* to all of media at 37°*C*. After 48 and 72 *hr* of incubation time, the lipase activity decreased in all of low and high cost carbon sources. This could be related to the consumption of nutritional elements in media and also production of protease enzyme simultaneous lipase under shack flask condition ^17^.

It is demonstrated that lipases are inducible enzymes that sources such as carbon could promote their gene expression. In many studies, for obtaining a high lipase enzyme yield, the effect of many low and high cost carbon sources including glucose, sucrose, mannitol, arabinose, lactose and olive, corn, soy, sunflower and maize oils, soybean meal and polyurethane were tested by one variable at a time and/or response surface methodology. One of the remarkable points in most of the studies was the fact that higher lipase activity is produced in the presence of lipidic carbon sources such as oils and fatty acids ^2,7,18–21^. Olive and mustard oil and tween 80 act as a carbon source and inducer had shown the highest lipase activity for *Bacillus mycoides* and *Bacillus coagulans,* respectively ^22,23^.

Several studies suggest that yeast extract provided amino acids, peptides, vitamins and carbohydrate which are necessary for rapid growth of microorganisms ^24^. In many cases, peptone and tryptone are known as a common inducer for lipase and protease production. The releasing of NH_4_^+^ ions from peptone influenced its efficacy for higher enzyme activity because NH_4_^+^ stimulates the growth and increases enzyme production rate ^2^. The best result for maximum production of lipase was obtained in the presence of peptone, yeast extract as organic nitrogen source and NH_4_NO_3_ in different bacterial strains ^20,25,26^. In other studies, beef extract and yeast extract were found as a nitrogen source for lipase production by *Bacillus pumilus B106* and *Bacillussp*., respectively ^27,28^. Among nitrogen sources, fish powder was selected in this study and it showed great influence on promotion of lipase expression after 24 *hr*.

It is established that for industrial production of lipase enzyme, less incubation time is favorable. It is important to note that in all conditions which were tested in this work, the best level of acidity was shown in 24 *hr* after incubation. Other factors in culture media could affect lipase production and its activity such as pH, salt, inoculum size, temperature and etc. The results of these studies demonstrated that culture conditions have an important role in enzyme production ^12,13,18,19,27,28^.

## Conclusion

It is well known that high enzyme productivity has been obtained by culture media optimization. For achieving this goal, there are important variables which many researchers focused on including substrates, inducers, nitrogen and carbon sources for increasing lipase production. For commercial purpose and on industrial scale, lipase enzymes production, generally low cost sources, was selected. Use of low cost sources could lead to reduction in costs for large scale processes. To achieve this important goal, the optimum lipase activity was found by *Bacillus sp. ZR-5* after 24 *hr* of incubation by 1.5% molasses, 1% fish powder and 2% olive oil. On the basis of our results, it can be suggested that these conditions for industrial production of lipase enzyme are suitable.
